# Hyperspectral imaging of foodborne pathogens at colony and cellular levels for rapid identification in dairy products

**DOI:** 10.1002/fsn3.3766

**Published:** 2023-10-13

**Authors:** Amninder Singh Sekhon, Phoebe Unger, Sonali Sharma, Bhupinderjeet Singh, Xiongzhi Chen, Girish M. Ganjyal, Minto Michael

**Affiliations:** ^1^ School of Food Science Washington State University Pullman Washington USA; ^2^ Biological Systems Engineering Department Washington State University Pullman Washington USA; ^3^ Department of Mathematics and Statistics Washington State University Pullman Washington USA

**Keywords:** dairy, foodborne pathogens, hyperspectral imaging, rapid identification

## Abstract

This study evaluated the efficacy of hyperspectral imaging (HSI) for the rapid identification of pathogens in dairy products at the colony and cellular levels. The colony and cellular levels studies were designed as completely randomized with six replications. Three strains of *Listeria monocytogenes*, four strains of *Escherichia coli* O157: H7, Big Six Shiga toxin‐producing *E. coli*, three strains of *Staphylococcus aureus*, and ten serovars of *Salmonella* were used in this study. Pure cultures were streaked for isolation on respective selective media, and hyperspectral data (400–1100 nm wavelength) at the colony and cellular levels were collected and stored as reference libraries. Whole milk and whole milk powder were artificially inoculated (<10 CFU/g or mL) with individual pathogenic strains/serovars. All milk and milk powder samples were enriched using brain heart infusion (BHI) broth at 37°C for 24 h, streaked for isolation on the respective selective media, and hyperspectral data for individual pathogenic strains/serovars at the colony and cellular levels were acquired and treated as test samples data. The acquired colony or cellular images were imported into ENVI software and three regions of interest were selected for each image to obtain hyperspectral data for reference libraries and test samples. Using the *k*NN classifier and cross‐validation technique, overall classification accuracies of 90.38% and 34% were obtained for the colony‐ and cellular‐level identification, respectively. The individual classification accuracies of pathogens in dairy products at the colony level varied between 77.5% to 100%, whereas the accuracy varied between 2.78% and 49.17% for the cellular level.

## Graphical abstract

The overall classification accuracy of 90.38% of the studied HSI system with the *k*NN classification model for various pathogens at the colony level supports the hypothesis that HSI can be used as a rapid pathogen identification tool in the food industry. However, this HSI system was not effective in accurately identifying various pathogens at the cellular level. To improve the classification accuracy of this HSI system at the colony level, more research should be conducted to improve the magnification of bacterial cells under the microscope.

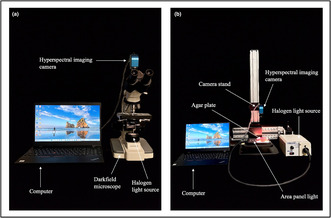

## 1 INTRODUCTION

Foodborne outbreaks and associated illnesses or deaths have consistently raised several questions about the existing food safety assessment parameters. As per an estimate from the Centers for Disease Control and Prevention (CDC), one in six Americans gets sick each year, which results in 128,000 hospitalizations and 3000 deaths (CDC, 2018). In the United States, an estimated 760 illnesses and 22 hospitalizations per year are linked to the consumption of dairy products, mainly caused by Shiga‐toxin‐producing *Escherichia coli* (STEC), *Salmonella*, *Listeria monocytogenes*, and *Campylobacter* (Costard et al., 2017). The increased number of foodborne outbreaks has reinforced the need for a rapid pathogen identification system. Although conventional microbiological detection methods are accurate and considered the gold standard, these methods are laborious, time‐consuming, and take 4–7 days to produce results (Park et al., 2012). The presently employed rapid detection methods (immunological or molecular based) require complex instrumentation, intensive sample preparation, costly reagents, and trained individuals for operation and maintenance. Therefore, a rapid, accurate, sensitive, and cost‐effective pathogen identification system is required to detect pathogens at an early stage.

Hyperspectral imaging (HSI) is a novel technology in microbial identification, which is rapid, reliable, non‐destructive, and requires minimal sample preparation. The HSI technology combines conventional imaging and spectroscopy that can simultaneously acquire spatial (x and y dimensions of an image) and spectral features (wavelength, λ) of a specimen (Gowen et al., 2007). In HSI, the specimen images are usually collected in the visible/near‐infrared region (400–1000 nm) at specific wavelength intervals. The acquired spatial and spectral information is combined to form a dataset known as a hypercube (x, y, λ). Depending on the molecular and chemical structure of a sample, the hypercube can consist of absorption, reflectance, transmittance, or fluorescence spectral data. The hypercube contains a stack of two‐dimensional images as a function of a given wavelength range, where each pixel of an image encompasses its own complete spectrum or unique hyperspectral signature (Wu & Sun, 2013). Depending on the research type, a pixel or group of pixels [known as the region of interest (ROI)] can be selected from a hypercube to generate the hyperspectral signature. This hyperspectral signature is a unique fingerprint for rapidly identifying a given specimen. For example, the hyperspectral signature of a given bacterial colony or cell can be stored in a reference library and can be utilized later to identify unknown samples by matching the hyperspectral signatures.

The HSI is widely utilized in the military (Goetz et al., 1985), astronomy (Hege et al., 2004), agriculture (Monteiro et al., 2007), pharmaceuticals (Lyon et al., 2002), and medical science (Muselimyan et al., 2016). In the field of food quality, the HSI has been studied for assessing the quality of fruits and vegetables (Qin & Lu, 2005; Vargas et al., 2005), meat (Naganathan et al., 2008; Qiao et al., 2007), fish (Heia et al., 2007), and grains (Pearson et al., 2001). Regarding food safety, a considerable amount of research has been performed at the macrolevel (i.e., colony level), and the use of HSI at the single bacterial cell level still needs to be explored. In terms of bacterial identification at the colony level, the HSI has been studied for differentiating Big Six non‐O157 STEC (O26, O45, O103, O111, O121, and O145) colonies using pure and mixed cultures with a classification accuracy of 84 to 100% (Yoon et al., 2013a, 2013b). Yoon et al. (2010) also used HSI to differentiate *Campylobacter* colonies from other contaminants with a classification accuracy of 99%.

Commercially available pre‐assembled and pre‐programmed HSI systems are costly, which impedes further exploring this technology. Therefore, it is imperative to develop a cost‐effective and reliable HSI system to encourage future research in the food safety arena. In our previous research, we developed a cheaper version of the HSI system by mounting a snapshot hyperspectral imaging camera over a dark‐field microscope (Unger et al., 2022). This custom‐built HSI system achieved a classification accuracy of 58.97% and 61.53% for *E. coli* O157:H7 and *L. monocytogenes*, respectively, at the cellular level.

Therefore, in this research, we further investigated the efficacy of our HSI (both at colony and cellular levels) by using a total of 26 strains or serovars of four different foodborne pathogens. The bacteria used in this study included *E. coli* (O157:H7 and Big Six STEC), *L. monocytogenes*, *Salmonella*, and *Staphylococcus aureus*. The primary aim of this research was to obtain hyperspectral signatures (both at colony and cellular levels) of the common foodborne pathogens for building standard reference libraries and to subsequently evaluate the efficacy of the HSI system to rapidly identify the different pathogens in the artificially inoculated dairy products (whole milk and whole milk powder).

## 2 MATERIALS AND METHODS

### 2.1 Hyperspectral imaging system

**FIGURE 1:**
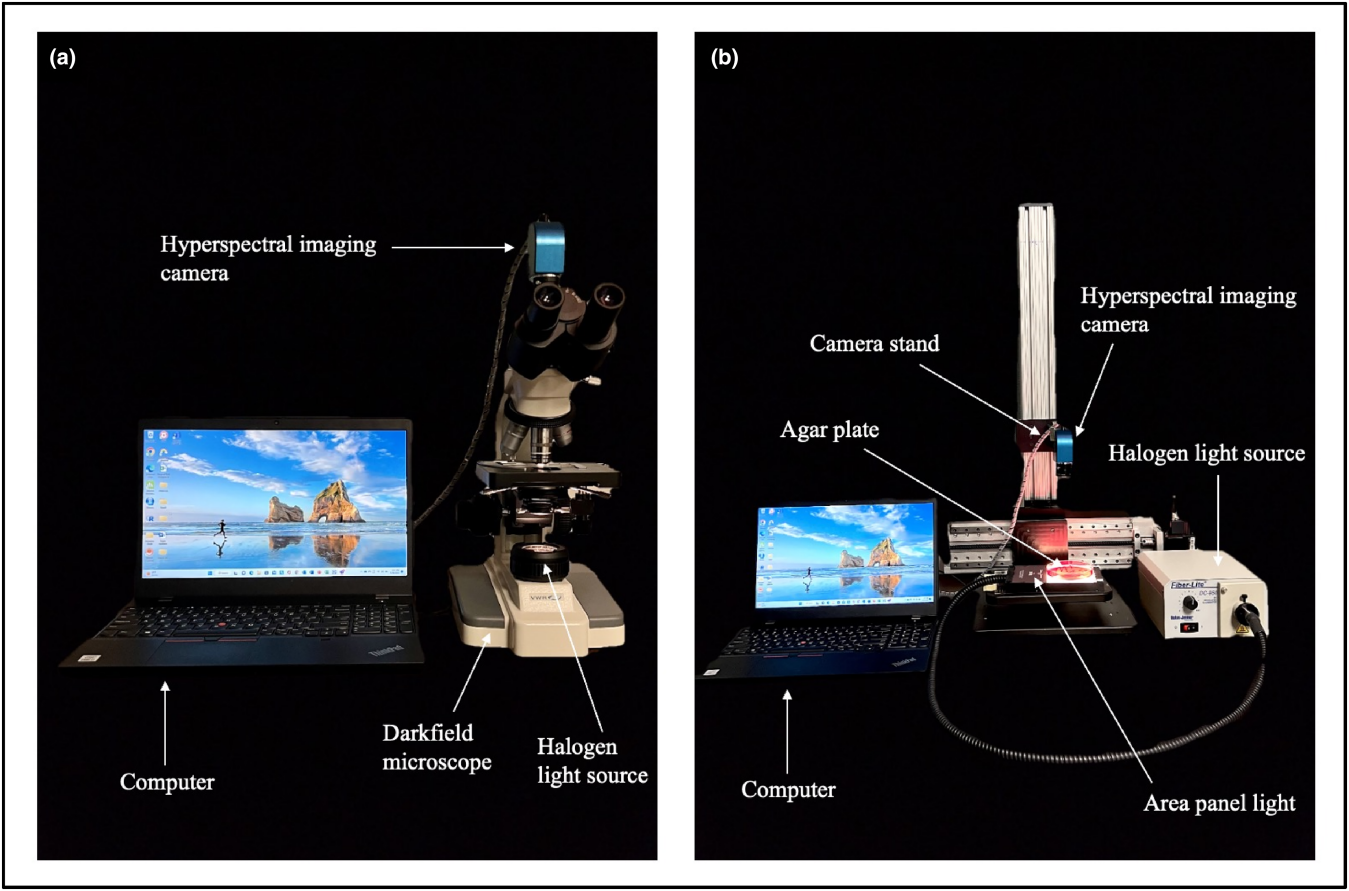
Hyperspectral imaging system for acquiring hyperspectral data at (a) cellular level and (b) colony level.

A custom‐assembled HSI system used in this research for acquiring hyperspectral data at the cellular level has been explained in detail by Unger et al. (2022). Briefly, a GoldenEye™ snapshot hyperspectral imager (BaySpec Inc., San Jose, CA) was mounted over a B3‐223 trinocular dark‐field microscope (VWR® International, Radnor, PA) through engineering adjustments to integrate these two technologies into a functional system (Figure 1a). The hyperspectral imager was connected through a USB port to a computer (Lenovo®, Morrisville, NC). However, the HSI system used for acquiring hyperspectral data at the colony level was similar to the system used for acquiring the cellular level data but without a microscope (Figure 1b). This HSI system consisted of a hyperspectral imager mounted on a camera stand, a computer to control the camera and obtain images, and a Fiber‐Lite DC950 illuminator halogen light source (Setra Systems, Inc., Boxborough, MA) connected with fiber optic area panel light (Setra Systems, Inc., Boxborough, MA) to illuminate the agar plate from the bottom (Figure 1b). During image acquisition, cellular‐ or colony‐level imaging systems were covered with specially designed black cardboard boxes to eliminate any background noise.

### 2.2 Experimental design

The colony and cellular levels studies were considered as two independent experiments, where each study was designed as a completely randomized design with six replications (i.e., work with each strain was replicated six times). A total of 26 strains/serovars of known pathogens [*E. coli* (O157:H7 and Big Six non‐O157 STEC), *L. monocytogenes*, *Salmonella*, and *S. aureus*] were used to collect hyperspectral data at the colony or cellular level. The hyperspectral signatures of immobilized cells (cellular level) or isolated colonies on the agar plates (colony level) were acquired for each replication. Three pathogenic cells or colonies from a captured hyperspectral image were selected as ROI to generate the hyperspectral graphs. For a given pathogenic strain/serovar, mean scattering intensity values of three ROIs were used to generate hyperspectral graphs at wavelengths ranging from 400 to 1100 nm. The hyperspectral signatures of pathogens were used to generate the hyperspectral reference libraries at cellular and colony levels to train the classification model. The *k*‐nearest‐neighbor (*k*NN) classifier (with an optimal *k* determined using cross‐validation) and hierarchical clustering were utilized to classify the unknown pathogenic cells or colonies from artificially inoculated dairy products (milk or milk powder) using the software R version 4.2.2 (The R Foundation for Statistical Computing, Vienna, Australia). The hyperspectral signatures of bacterial cells or colonies from pure cultures or artificially inoculated food matrices (milk and milk powder) were acquired randomly within each replication.

### 2.3 Culture propagation

The bacterial cultures used in this study are presented in Table 1. All pathogenic strains/serovars were selected based on their food safety risk and involvement in foodborne disease outbreaks. Freeze‐dried cultures of the individual strains/serovars were propagated as per the manufacturer's instructions and then transferred onto glycerol protectant beads (Microbank™, Richmond Hill, ON) and stored at −80°C freezer (Panasonic Healthcare Co., Ltd., Wood Dale, IL). A frozen bead of each pathogen was grown individually at 37°C for 24 h in 10 mL of brain heart infusion (BHI) broth (Difco™, Becton, Dickinson and Company, Sparks, MD) and stored at 4°C as stock cultures. The stock cultures of *Salmonella* and *E. coli* were confirmed using API® 20E (bioM'erieux, Inc., Durham, NC), whereas *L. monocytogenes* and *S. aureus* were confirmed using API® *Lister* and API® *Staph* (bioM'erieux, Inc., Durham, NC), respectively.

**TABLE 1:** Pathogenic strains/serovars used in this research study.

Pathogen	Serovar/strain	ATCC^a^ No.
*Listeria monocytogenes*	–	5414
	–	19,111
	–	19,115
*Staphylococcus aureus*	–	13,565
	–	14,458
	–	51,560
*Salmonella*	Agona	BAA‐707
	Enteritidis	BAA‐708
	Heidelberg	8326
	Infantis	51,741
	Mbandaka	51,958
	Montevideo	BAA‐710
	Newport	6962
	Senftenberg	43,895
	Typhi	33,459
	Typhimurium	14,028
*Escherichia coli* O157:H7	–	Non‐ATCC^b^
	–	12,900
	–	35,150
	–	43,895
Big Six non‐O157 STEC^c^	O26	BAA‐2196
	O45	BAA‐2193
	O103	BAA‐2215
	O111	BAA‐2217
	O121	BAA‐2219
	O145	BAA‐2129

^a^ American Type Culture Collection (ATCC®, Manassas, VA).

^b^ University of Idaho, Moscow, ID (Sheng et al., 2006).

^c^ Shiga toxic‐producing *Escherichia coli*.

### 2.4 Sample preparation using selective media

For each replication, a loop from an individual stock culture was used to inoculate 10 mL of BHI broth and incubated at 37°C for 24 h. Freshly propagated individual cultures of respective pathogens were streaked for isolation on the respective selective media. The PALCAM (Difco™, Becton, Dickinson and Company, Sparks, MD), Sorbitol MacConkey agar (Hardy diagnostics, Santa Maria, CA), R&F non‐O157 STEC chromogenic media (R&F products, Downers Grove, IL), Xylose Lysine Deoxycholate (XLD) agar (Himedia laboratories Pvt. Ltd., Dindari, Nashik), and Baird Parker agar (Difco™, Becton, Dickinson and Company, Sparks, MD) were used as selective media for *L. monocytogenes*, *E. coli* O157:H7, Big Six non‐O157 STEC, *Salmonella*, and *S. aureus*, respectively. For the cellular study, a minute part of a single colony of a respective pathogen was mixed in 1 mL of filtered (0.2 μm) sterile HPLC‐grade water (J.T. Baker Inc., Phillipsburg, NJ) and vortexed for 1 minute. Subsequently, using a sterile loop (0.01 mL) (VWR® International, Radnor, PA), the vortexed solution was transferred onto a 1 mm glass slide (Fisherbrand, Fisher Scientific, Pittsburgh, PA). The bacterial cells were immobilized by ambient air drying the slide in a biosafety cabinet for 5 min. The slide was then analyzed using the HSI system (Figure 1a). For the colony study, the agar plates with isolated colonies were analyzed directly under the HSI system (Figure 1b).

### 2.5 Milk and milk powder inoculation

Whole milk powder and whole milk are key ingredients in numerous food products. Therefore, in this research, whole milk and whole milk powder were selected as inoculation media to represent high water activity (a_w_) and low a_w_ foods, respectively. Moreover, research aimed to validate the efficacy of custom‐assembled HSI system for rapid identification of pathogens in dairy products; therefore, whole milk powder (Hoosier Hill Farms, Fort Wayne, IN) and whole milk (reconstituted from whole milk powder @ 13% w/v) were chosen as dairy products. The milk and milk powder were artificially inoculated with the respective pathogenic strain/serovar so that their population was less than 10 CFU/mL or g. Freshly propagated individual pathogenic cultures were prepared from stock cultures as mentioned previously, and then serially diluted using 9 mL of 0.1% peptone solution to prepare the final inoculum (Bacto™, Becton, Dickinson and Company, Sparks, MD). Thereafter, milk powder (9 g) was mist inoculated using a spray nozzle, which was calibrated to spray ~1 mL of inoculum per squirt. Approximately, 1 mL of inoculum was used to inoculate the milk powder. Similarly, 9 mL of reconstituted milk was inoculated using 1 mL of the inoculum. The inoculated whole milk powder or whole milk was spread and plated on respective selective agars for enumeration to confirm the bacterial population (<10 CFU/mL). To simulate the real‐life situation where bacterial cells can be present in low numbers and can fall below the detection limit, the milk and milk powder samples were enriched by mixing 1 mL or 1 g of inoculated milk or milk powder with 9 mL of BHI broth and incubating at 37°C for 24 h. After enrichment, the individual pathogenic strains/serovars were streaked for isolation on the respective selective media and incubated at 37°C for 24 h. Then, the HSI analysis was conducted for the colonies or cells from the inoculated milk and milk powder samples, as described in the previous section.

### 2.6 Generation of hyperspectral graphs

The Environment for Visualizing Images (ENVI) (Harris Geospatial Solutions Inc., Boulder, CO) software version 5.6 was employed for generating hyperspectral graphs from the acquired hyperspectral images at the colony or cellular level. Hyperspectral images of individual bacterial cells (air‐dried on a glass slide) were obtained by focusing the microscope at 2000× magnification, and ENVI settings at 0 gain and 50 ms exposure time. The snapshot hyperspectral technique was used to capture the hyperspectral images. The snapshot technique can simultaneously acquire spatial and spectral data within a single exposure without the movement of the sample or detector. From an acquired hyperspectral image at the cellular level, three bacterial cells were selected as ROI. The average scattering intensity values of three ROI were used to generate hyperspectral graphs at wavelengths ranging from 400 to 1100 nm (at wavelength intervals of 5 nm resulting in 141 wavelength bands). For colony‐level imaging, the exact same software settings were employed, except the hyperspectral camera was attached to a camera stand (without a microscope), and the Fiber‐Lite DC 950 illuminator halogen light source connected with fiber optic area panel light was used to illuminate the agar plates from the bottom.

For constructing the reference libraries at cellular and colony levels for the 26 pathogenic strains/serovars, the hyperspectral data were acquired by conducting six independent replications for all cultures. Therefore, data from 156 images (i.e., 6 replications for 26 strains/serovars) at colony or cellular levels were stored as reference libraries to train the optimal *k*NN classification models separately for colony and cellular data. Similarly, for a given food matrix (whole milk or whole milk powder), 156 images of known strains/serovars were acquired at colony or cellular, which were used to test against the training data from the reference libraries. Therefore, a total of 468 images were acquired at wavelengths ranging from 400 to 1100 nm.

### 2.7 *k*NN classification and validation of hyperspectral graphs

The hyperspectral graphs for this research were pre‐processed by normalizing the scattering intensity values at y‐axis values from 0 to 1 (Michael et al., 2019; Unger et al., 2022). The values “0” and “1” were considered the darkest and brightest points on the ROI, respectively. The normalized scattering values were calculated using the following equation (Scott et al., 2006).

$$y_{\mathrm{ij}}=\left[x_{\mathrm{ij}}-\min\;\left(X_{j}\right)\right]/\left[\max\;\left(X_{j}\right)-\min\;\left(X_{j}\right)\right]$$

where *X*_*j*_ is a numeric vector and is the hyperspectral signature of the *j*th observation, *x*_*ij*_ the *i*th entry of *X*_*j*_ and is the scattering value at the *i*th wavelength, min (*X*_*j*_) is the minimum scattering value of the hyperspectral signature *X*_*j*_, and max (*X*_*j*_) is the max scattering value of the hyperspectral signature *X*_*j*_.

The normalized hyperspectral signatures from individual pathogens were classified using the *k*NN classifier. The *k*NN is a non‐parametric classification algorithm that determines the class of an unknown data point by looking at the classes of the nearest known data points. The number *k* determines how many nearest neighbors will be considered. The Euclidean pairwise distance was calculated to compute the dissimilarity between a pair of normalized hyperspectral signatures. To decide an optimal neighboring size *k*, a training set of 156 normalized hyperspectral signatures of known pathogens was created, and a fourfold cross‐validation was applied to the training data set. This gave an optimal value of *k* as 3 for the colony data, whereas for the cellular level, the optimal *k* was 12. For this study, fourfold cross‐validation was selected to estimate the test error of an optimal *k*NN classifier. The optimal 3‐NN (colony level) or 12‐NN (cellular level) classifier was then applied to classify a total of 312 normalized hyperspectral signatures (test data set) from dairy products, all distinct from those in the training set, to classify them into their corresponding pathogens.

### 2.8 Hierarchical clustering analysis

The hierarchical clustering analysis (HCA) is an unsupervised machine learning algorithm that groups similar data points into clusters, where each cluster is further divided into smaller sub‐clusters until each data point forms its own cluster. The hierarchy of clusters can be depicted using a tree‐like diagram called a dendrogram. The clustering process begins with each data point being its cluster, and then it repeatedly combines the closest pairs of clusters until only one big cluster remains or until a stopping criterion is met (Nielsen, 2016). For clustering analysis, mean hyperspectral signatures of the 26 pathogenic strains/serovars from the reference libraries at cellular and colony levels were utilized. The R function “hclust” performed the clustering analysis. Initially, each pathogen is considered an individual cluster. After that, the most similar pathogens or mean hyperspectral signatures are merged into a new cluster until all pathogens belong to one cluster. The similarities or dissimilarities between the mean hyperspectral signatures of the pathogens are represented by the dendrogram tree. For this analysis, we first calculated the pairwise distance using the Euclidean distance metric to measure the similarity between the pathogens. Further clustering was performed using linkage criteria which depends on pairwise distances calculated earlier (Buchgraber et al., 2004). To merge the clusters, a single linkage method was employed. The single linkage method calculates all pairwise distances between the observations in cluster A and the observations in cluster B and records the shortest of these distances to merge clusters.

## 3 RESULTS

**FIGURE 2:**
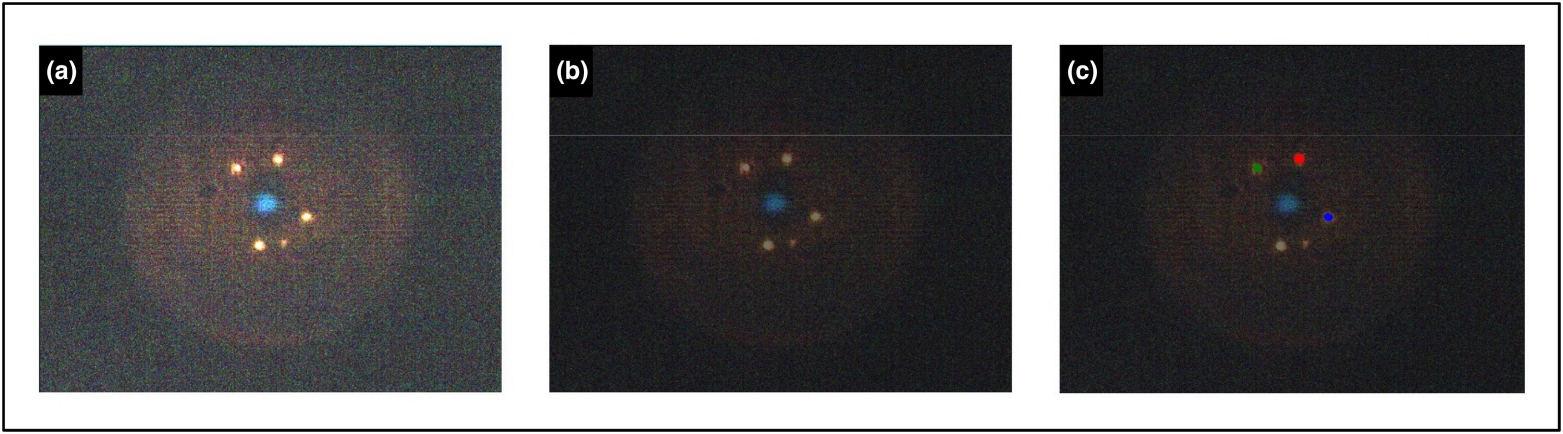
Hyperspectral images of *Salmonella* Typhimurium cells on glass slide at 2000× magnification: (a) Hyperspectral image as it appears when imported in the Environment for Visualizing Images (ENVI); (b) Hyperspectral image after pre‐processing technique has been applied in ENVI; (c) Hyperspectral image with three regions of interest (ROIs) selected (green, red, and blue) in ENVI.

**FIGURE 3:**
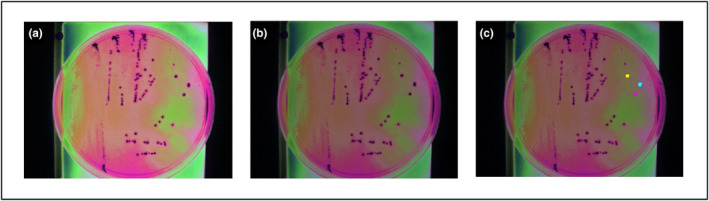
Hyperspectral images of *Salmonella* Typhimurium colonies on xylose lysine deoxycholate (XLD) agar plate: (a) Hyperspectral image when imported in Environment for Visualizing Images (ENVI); (b) Hyperspectral image after pre‐processing technique has been applied in ENVI; (c) Hyperspectral image with three regions of interest (ROIs) selected (yellow, pink, and cyan) in ENVI.

Figures 2a and 3a display the hyperspectral images of bacterial cells and colonies, respectively, as they appear when imported into ENVI software. The images were clarified using the ENVI software, which did not affect the hyperspectral signatures (Figures 2b and 3b). The selection of the individual bacterial cells or colonies as ROI is presented in Figures 2c and 3c, respectively.

**FIGURE 4:**
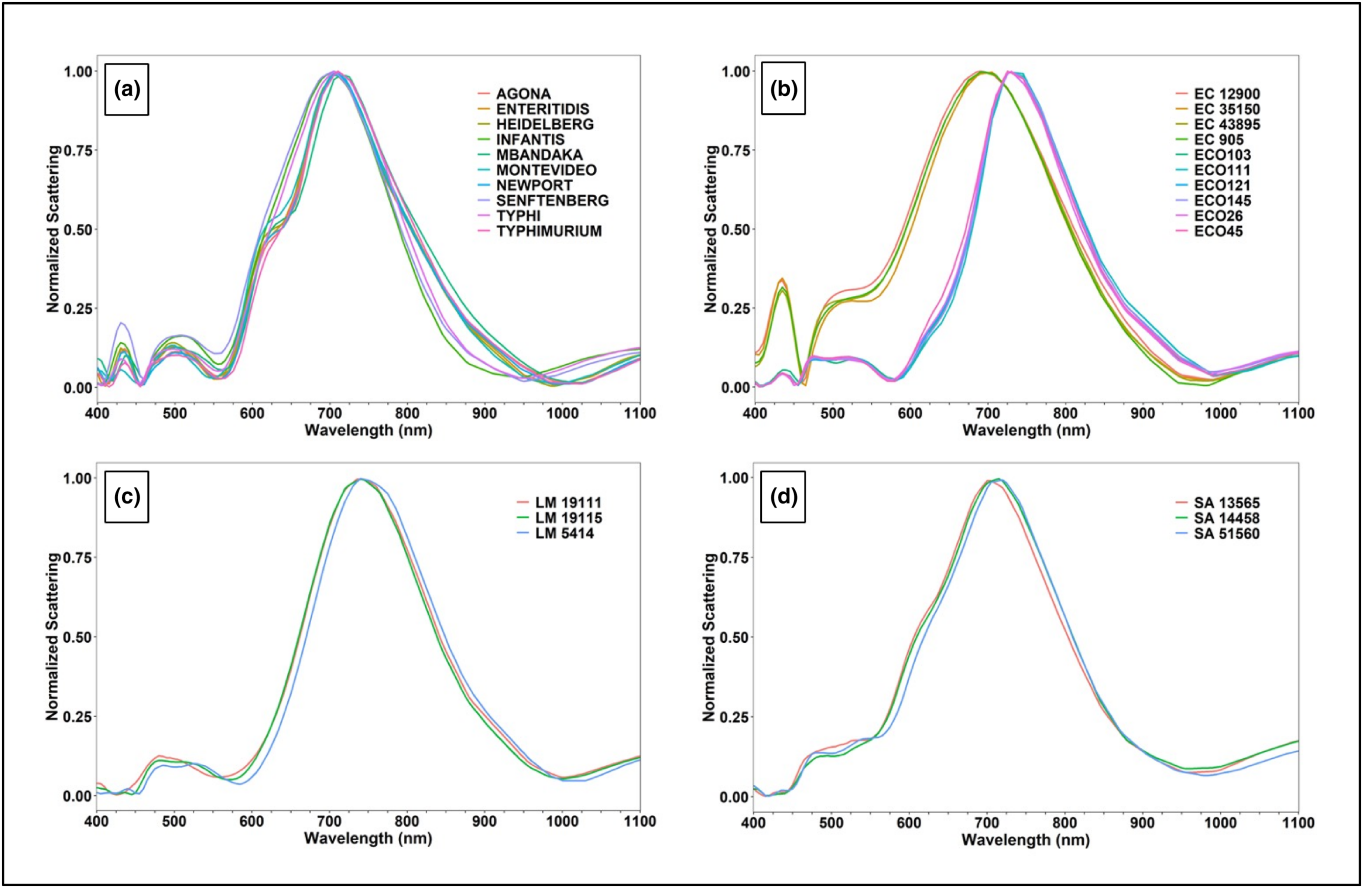
Mean normalized hyperspectral graphs of the reference library of (a) *Salmonella* serovars; Agona, Enteritidis, Heidelberg, Infantis, Mbandaka, Montevideo, Newport, Senftenberg, Typhi, and Typhimurium; (b) *Escherichia coli* (EC) O157:H7 strains 905, 35,150, 43,895, and 12,900, and Big Six non‐O157:H7 serovars O26, O45, O103, O111, O121, and O145; (c) *Listeria monocytogenes* (LM) strains 19,111, 19,115, and 5414; and (d) *Staphylococcus aureus* (SA) strains 13,565, 14,458, and 51,560 captured at wavelengths ranging from 400 to 1100 nm (with 5 nm band intervals) at the colony level.

Figure 4 represents the mean normalized scattering intensities of the 26 pathogenic strains/serovars from the reference library at a colony level, which demonstrates the differences in the scattering intensities or hyperspectral signatures of these strains/serovars within their respective genera. For example, the scattering intensity values for all *Salmonella* serovars were noticeably different for wavelengths ranging from 400 to 450 nm, 475 to 700 nm, and 750 to 1100 nm (Figure 4a). The hyperspectral signatures of *E. coli* O157:H7 strains (905, 12,900, 35,150, and 43,895) differ from the Big Six non‐O157 STEC serovars (Figure 4b). Additionally, the hyperspectral signatures were different within O157:H7 strains and within Big Six non‐O157 STEC serovars. The main differences within O157:H7 strains can be observed between 400 and 450 nm, 475 and 675 nm, and 875 and 1000 nm; however, for Big Six non‐O157 STEC serovars, the differences were discernable between 425 and 525 nm, 600 and 675 nm, and 850 and 975 nm (Figure 4b). The hyperspectral signatures for strains of *L. monocytogenes* (5414, 19,111, and 19,115) were noticeably different between 400 and 625 nm and 750 and 1000 nm (Figure 4c). Likewise, for *S. aureus*, the differences in the scattering intensity values for strains 13,565, 14,458, and 51,560 were observable between 450 and 750 nm and 925 and 1025 nm (Figure 4d).

**FIGURE 5:**
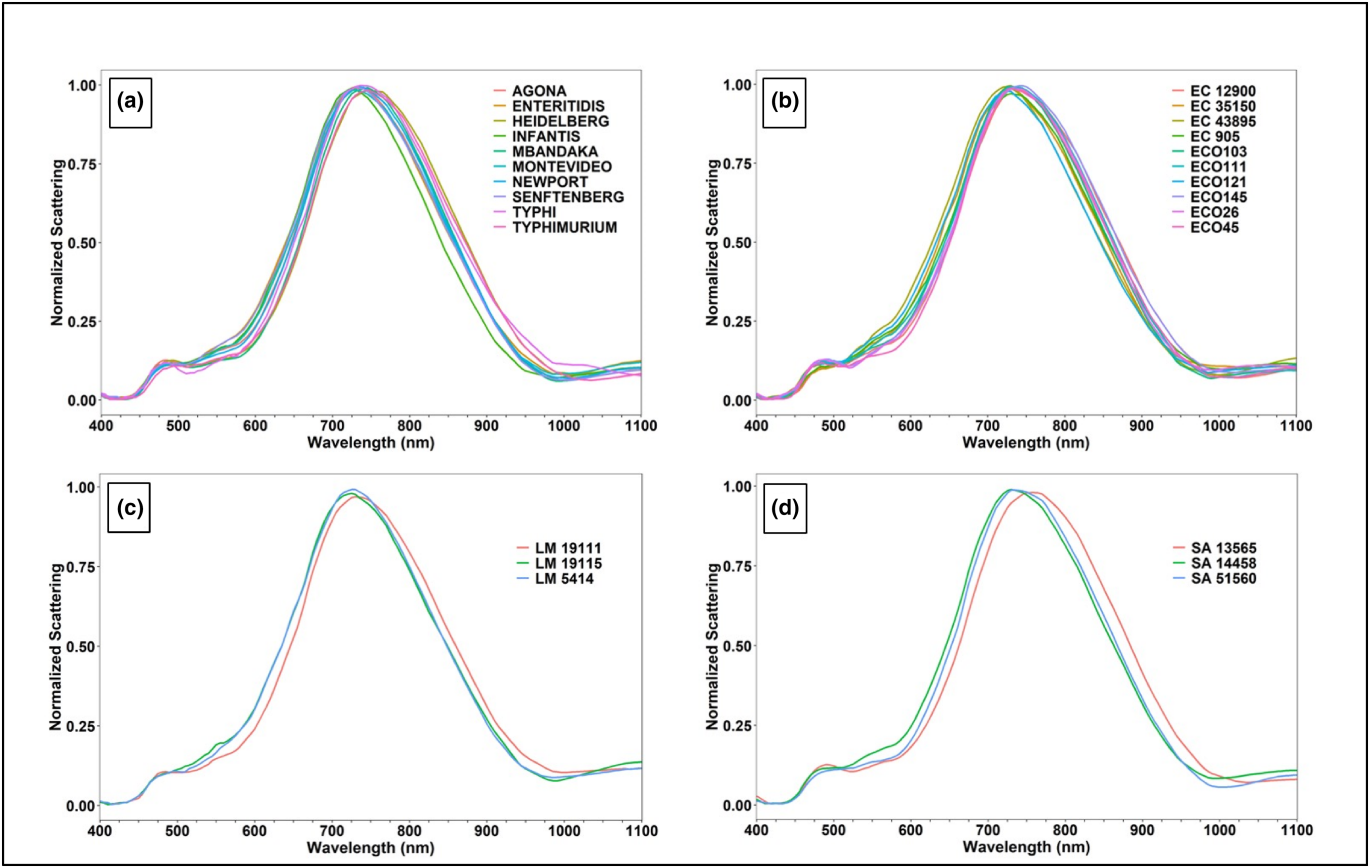
Mean normalized hyperspectral graphs of the reference library of (a) *Salmonella* serovars Agona, Enteritidis, Heidelberg, Infantis, Mbandaka, Montevideo, Newport, Senftenberg, Typhi, and Typhimurium; (b) *Escherichia coli* (EC) O157:H7 strains 905, 35,150, 43,895, and 12,900, and Big Six non‐O157:H7 serovars O26, O45, O103, O111, O121, and O145; (c) *Listeria monocytogenes* (LM) strains 19,111, 19,115, and 5414; and (d) *Staphylococcus aureus* (SA) strains 13,565, 14,458, and 51,560 captured at wavelengths ranging from 400 to 1100 nm (with 5 nm band intervals) at the cellular level.

Figure 5 represents the mean normalized scattering intensities of strains/serovars of the 26 pathogens from the reference library at a cellular level. At a cellular level, a high degree of overlap for hyperspectral signatures of 26 pathogens was observed. For example, most strains/serovars had peak scattering intensities between 700 and 750 nm wavelengths, and similar scattering patterns were observed between 400 and 500 nm range for different strains/serovars within each pathogen type (Figure 5a–d). The different strains/serovars within *Salmonella*, *E. coli*, and *S. aureus* demonstrated notable differences between 500 and 1100 nm (Figure 5a,b,d). However, strains of *L. monocytogenes* displayed major differences in the scattering patterns between 500 and 575 nm, 700 and 800 nm, and 875 and 1100 nm (Figure 5c).

**FIGURE 6:**
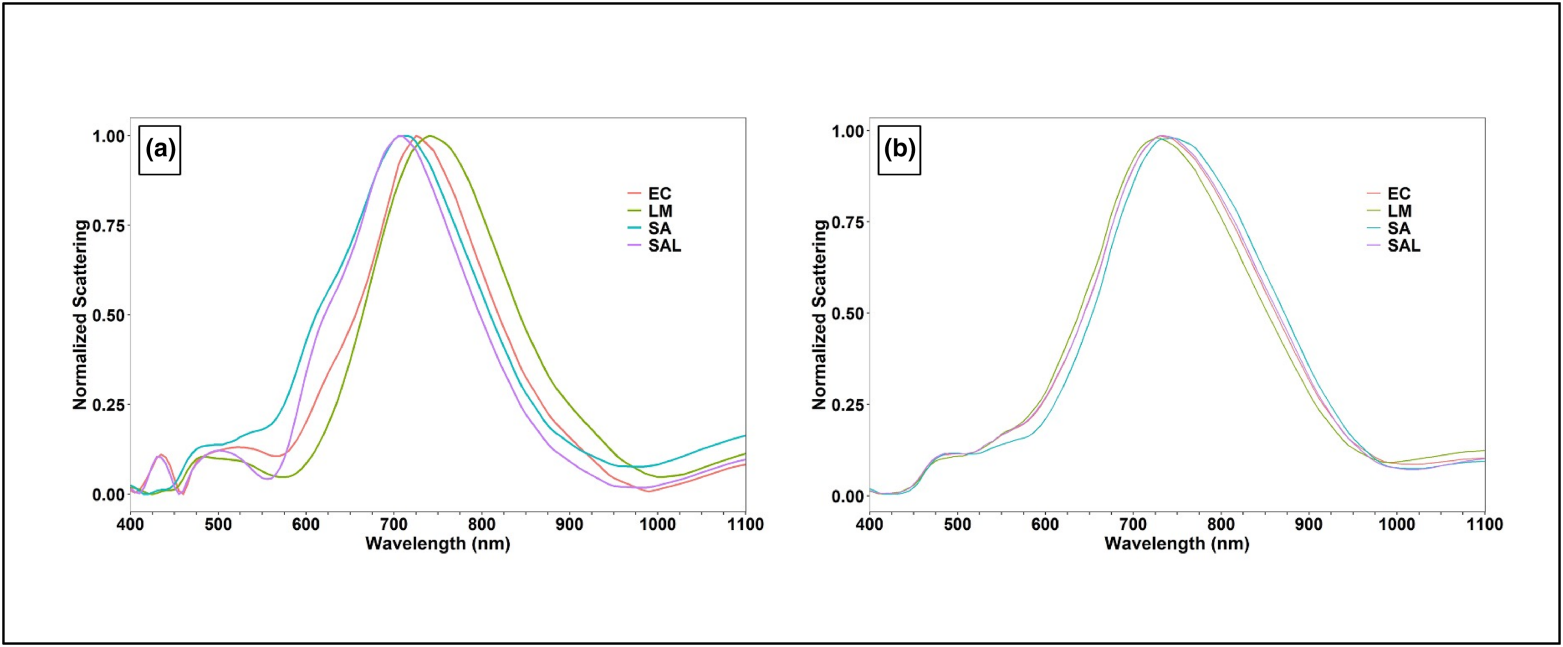
Mean normalized hyperspectral graphs^†^ of the reference libraries of *Escherichia coli* (EC), *Listeria monocytogenes* (LM), *Staphylococcus aureus* (SA), and *Salmonella* (SAL) captured at a wavelength ranging from 400 to 1100 nm (with 5‐nm band intervals) at (a) colony level and (b) cellular level. ^†^For each individual pathogen (EC, LM, SA, or SAL), the mean hyperspectral signatures for their respective strains/serovars were averaged to obtain a single spectral signature.

Figure 6a,b present the mean normalized hyperspectral signatures of *Salmonella*, *L. monocytogenes*, *E. coli*, and *S. aureus* from the reference library at colony and cellular levels, respectively. These mean hyperspectral signatures were obtained by averaging all strains/serovars within the respective genus. The hyperspectral signatures for different genera at the colony level were clearly distinguishable from each other, and different pathogens had noticeably different peak scattering intensity values ranging between 700 and 750 nm (Figure 6a). On the other hand, at a cellular level, the hyperspectral signatures of *E. coli* and *Salmonella* exhibited significant overlapping, but the differences in the hyperspectral signatures of *L. monocytogenes* and *S. aureus* were discernable (Figure 6b). The peak scattering intensities at the cellular level for different pathogens ranged from 725 to 750 nm (Figure 6b).

The confusion matrices used to describe the performance of the classification algorithm used in the study at the colony and cellular levels are presented in Tables 2 and 3, respectively. The rows in these matrices represent the predicted class and the columns represent the actual class. A total of 312 hyperspectral images were taken from the inoculated dairy products, that is, 156 from whole milk samples and 156 from whole milk powder samples. The classification accuracies at the colony level for *E. coli*, *L. monocytogenes*, *S. aureus*, and *Salmonella* were 77.5% (93 of 120 *E. coli* samples), 100% (36 of 36 *L. monocytogenes* samples), 100% (36 of 36 *S. aureus* samples), and 97.5% (117 of 120 *Salmonella* samples), respectively (Table 2). However, the classification accuracies at the cellular level were very low compared to that at the colony level. At the cellular level, the classification accuracies for *E. coli*, *L. monocytogenes*, *S. aureus*, and *Salmonella* were 49.17% (59 of 120 *E. coli* samples), 11.11% (4 of 36 *L. monocytogenes* samples), 2.78% (1 of 36 *S. aureus* samples), and 35% (42 of 120 *Salmonella* samples), respectively (Table 3). The overall classification accuracy for this HSI system at the colony level was 90.38%, whereas for the cellular level, the overall accuracy was 34%.

**TABLE 2:** Confusion matrix for optimal *k*NN classifier (*k* = 3) to classify 312 hyperspectral signatures of pathogens (26 strains/serovars × 2 dairy products × 6 replications) in whole milk and whole milk powder samples at colony level as *Escherichia coli* (O157:H7 and Big Six non‐O157:H7) (EC), *Listeria monocytogenes* (LM), *Salmonella* (SAL), and *Staphylococcus aureus* (SA) using normalized scattering intensity values acquired at wavelengths ranging from 400 to 1100 nm with 5 nm band intervals.

Predicted	Actual			
	EC	LM	SA	SAL
EC	93^a^	0	0	2
LM	5	36^b^	0	0
SA	1	0	36^c^	1
SAL	21	0	0	117^d^

^a^ 93 of 120 EC samples were classified correctly, i.e., 77.5% accuracy.

^b^ 36 of 36 LM samples were correctly classified, i.e., 100% accuracy.

^c^ 36 of 36 SA samples were correctly classified, i.e., 100% accuracy.

^d^ 117 of 120 SAL samples were correctly classified, i.e., 97.5% accuracy.

**TABLE 3:** Confusion matrix for optimal *k*NN classifier (*k* = 12) to classify 312 hyperspectral signatures of pathogens (26 strains/serovars × 2 dairy products × 6 replications) in whole milk and whole milk powder samples at cellular level as *Escherichia coli* (O157:H7 and Big Six non‐O157:H7) (EC), *Listeria monocytogenes* (LM), *Salmonella* (SAL), and *Staphylococcus aureus* (SA) using normalized scattering intensity values acquired at wavelengths ranging from 400 to 1100 nm with 5 nm band intervals.

Predicted	Actual			
	EC	LM	SA	SAL
EC	59^a^	13	18	60
LM	10	4^b^	1	10
SA	5	2	1^c^	8
SAL	46	17	16	42^d^

^a^ 59 of 120 EC samples were correctly classified, i.e., 49.17% accuracy.

^b^ 4 of 36 LM samples were correctly classified, i.e., 11.11% accuracy.

^c^ 1 of 36 SA samples was correctly classified, i.e., 2.78% accuracy.

^d^ 42 of 120 SAL samples were correctly classified, i.e., 35% accuracy.

The classification results from the optimal *k*NN (*k* = 3) classifier at the colony level for whole milk and whole milk powder samples are presented in Tables 4 and 5, respectively. For whole milk and whole milk powder samples, the classification accuracies at the colony level for strains of *L. monocytogenes* and *S. aureus* were 100%. Likewise, for *Salmonella* serovars (except Typhi), classification accuracies of 100% were achieved at the colony level for both dairy products. Whole milk samples' classification accuracies for Big Six non‐O157 STEC serovars (except O26) were 100%, whereas serovar O26 displayed 83.33% classification accuracy (Table 4). For whole milk powder samples, the classification accuracies for the *E. coli* O26, O121, and O145 were 83.33%, 83.33%, and 66.67%, respectively, but the *E. coli* O45, O103, and O111 achieved perfect classification accuracies of 100% (Table 5). For *E. coli* O157:H7 strains, the classification accuracies for inoculated dairy products were lower. For whole milk samples, the accuracy varied between 66.67 and 83.33%, whereas *E. coli* O157:H7 strain 905 achieved the lowest accuracy of 66.67% (Table 4). For whole milk powder samples, *E. coli* O157:H7 strains 43,895 and 35,150 had the worst classification accuracy of 16.67% (Table 5).

**TABLE 4:** Classification accuracies of various strains/serovars of *Escherichia coli* (O157:H7 and Big Six non‐O157), *Listeria monocytogenes*, *Salmonella*, and *Staphylococcus aureus* in whole milk samples at the colony level obtained from the optimal *k*NN (*k*‐nearest‐neighbor) classifier with optimal *k* = 3.

Food matrix	Bacteria	Strain/serovar	% classification accuracy	% Total classification accuracy
Whole milk	*Listeria monocytogenes*	5414	100	100
		19,111	100	
		19,115	100	
	*Staphylococcus aureus*	13,565	100	100
		14,458	100	
		51,560	100	
	*Salmonella*	Agona	100	98.33
		Enteritidis	100	
		Heidelberg	100	
		Infantis	100	
		Mbandaka	100	
		Montevideo	100	
		Newport	100	
		Senftenberg	100	
		Typhi	83.33	
		Typhimurium	100	
	*Escherichia coli* O157:H7	905	66.67	90
		12,900	83.33	
		35,150	83.33	
		43,895	83.33	
	Big Six non‐O157	O26	83.33	
		O45	100	
		O103	100	
		O111	100	
		O121	100	
		O145	100	

**TABLE 5:** Classification accuracies of various strains/serovars of *Escherichia coli* (O157:H7 and Big Six non‐O157), *Listeria monocytogenes*, *Salmonella*, and *Staphylococcus aureus* in whole milk powder samples at the colony level obtained from the optimal *k*NN (*k*‐nearest‐neighbor) classifier with optimal *k* = 3.

Food matrix	Bacteria	Strain/serovar	% classification accuracy	% Total classification accuracy
Whole milk powder	*Listeria monocytogenes*	5414	100	100
		19,111	100	
		19,115	100	
	*Staphylococcus aureus*	13,565	100	100
		14,458	100	
		51,560	100	
	*Salmonella*	Agona	100	96.67
		Enteritidis	100	
		Heidelberg	100	
		Infantis	100	
		Mbandaka	100	
		Montevideo	100	
		Newport	100	
		Senftenberg	100	
		Typhi	66.67	
		Typhimurium	100	
	*Escherichia coli* O157:H7	905	33.33	65
		12,900	50	
		35,150	16.67	
		43,895	16.67	
	Big Six non‐O157	O26	83.33	
		O45	100	
		O103	100	
		O111	100	
		O121	83.33	
		O145	66.67	

The classification results from the optimal *k*NN (*k* = 12) classifier at the cellular level for inoculated whole milk and whole milk powder samples are presented in Tables 6 and 7, respectively. The *E. coli* O157:H7 strains in dairy products achieved classification accuracies ranging from 33.33% to 66.67% at the cellular level. The *E. coli* O157:H7 strain 43,895 displayed the highest accuracy of 66.67%, whereas strain 905 had the lowest accuracy of 33.33% in dairy products (Tables 6 and 7). The classification accuracies for Big Six non‐O157 STEC serovars in whole milk samples varied from 33.33% to 83.33% (Table 6); however, for Big Six non‐O157 STEC serovars in whole milk powder samples, the classification accuracy was 33.33%, except strain O45 had 16.67% (Table 7). For *Salmonella* serovars in whole milk samples, the highest classification accuracy of 50% was achieved for the serovars Infantis and Enteritidis, but for other serovars, it ranged between 16.67% and 33.33% (Table 6). Likewise, for whole milk powder samples, *Salmonella* serovar Mbandaka had the highest classification accuracy of 66.67%, with other serovars displaying classification accuracies between 16.67% and 50% (Table 7). The strains of *L. monocytogenes* and *S. aureus* displayed poor classification accuracies for the inoculated dairy products. For whole milk and whole milk powder samples, the classification accuracies of *L. monocytogenes* strain 19,111, 19,115, and 5414 were 16.67%, 16.67%, and 0%, respectively. The *S. aureus* strains in dairy products had the worst classification accuracy of 0%, except *S. aureus* strain 14,458 in whole milk powder samples had 16.67% classification accuracy (Tables 6 and 7).

**TABLE 6:** Classification accuracies of various strains/serovars of *Escherichia coli* (O157:H7 and Big Six non‐O157), *Listeria monocytogenes*, *Salmonella*, and *Staphylococcus aureus* in whole milk samples at the cellular level obtained from the optimal *k*NN (*k*‐nearest‐neighbor) classifier with optimal *k* = 12.

Food matrix	Bacteria	Strain/serovar	% classification accuracy	% Total classification accuracy
Whole milk	*Listeria monocytogenes*	5414	0	11.11
		19,111	16.67	
		19,115	16.67	
	*Staphylococcus aureus*	13,565	0	0
		14,458	0	
		51,560	0	
	*Salmonella*	Agona	33.33	31.67
		Enteritidis	50	
		Heidelberg	16.67	
		Infantis	50	
		Mbandaka	33.33	
		Montevideo	16.67	
		Newport	33.33	
		Senftenberg	33.33	
		Typhi	33.33	
		Typhimurium	16.67	
	*Escherichia coli* O157:H7	905	50	58.33
		12,900	50	
		35,150	50	
		43,895	66.67	
	Big Six non‐O157	O26	66.67	
		O45	50	
		O103	83.33	
		O111	66.67	
		O121	66.67	
		O145	33.33	

**TABLE 7:** Classification accuracies of various strains/serovars of *Escherichia coli* (O157:H7 and Big Six non‐O157), *Listeria monocytogenes*, *Salmonella*, and *Staphylococcus aureus* in whole milk powder samples at the cellular level obtained from the optimal *k*NN (*k*‐nearest‐neighbor) classifier with optimal *k* = 12.

Food matrix	Bacteria	Strain/serovar	% Classification accuracy	% Total classification accuracy
Whole milk powder	*Listeria monocytogenes*	5414	0	11.11
		19,111	16.67	
		19,115	16.67	
	*Staphylococcus aureus*	13,565	0	5.56
		14,458	16.67	
		51,560	0	
	*Salmonella*	Agona	50	38.33
		Enteritidis	16.67	
		Heidelberg	33.33	
		Infantis	33.33	
		Mbandaka	66.67	
		Montevideo	33.33	
		Newport	33.33	
		Senftenberg	50	
		Typhi	50	
		Typhimurium	16.67	
	*Escherichia coli* O157:H7	905	33.33	40
		12,900	50	
		35,150	66.67	
		43,895	66.67	
	Big Six non‐O157	O26	33.33	
		O45	16.67	
		O103	33.33	
		O111	33.33	
		O121	33.33	
		O145	33.33	

**FIGURE 7:**
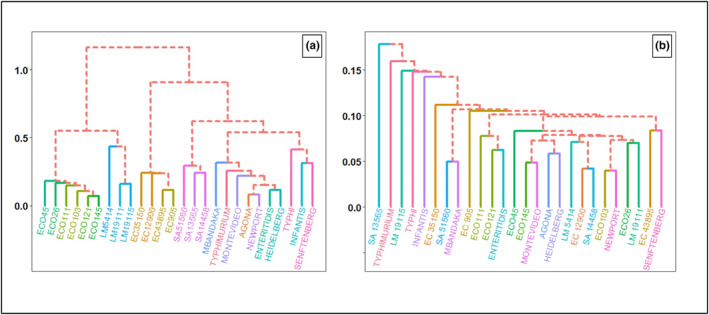
Hierarchical clustering dendrogram^†^ for *Salmonella* serovars (Agona, Enteritidis, Heidelberg, Infantis, Mbandaka, Montevideo, Newport, Senftenberg, Typhi, and Typhimurium), *Escherichia coli* (EC) O157:H7 strains (905, 35,150, 43,895, and 12,900), Big Six non‐O157 serovars (O26, O45, O103, O111, O121, and O145), *Listeria monocytogenes* (LM) strains (19,111, 19,115, and 5414), and *Staphylococcus aureus* (SA) strains (13,565, 14,458, and 51,560) captured at wavelengths ranging from 400 to 1100 nm (with 5 nm band intervals) at the (a) colony level and (b) cellular level. ^†^The dendrogram for the colony or cellular data was constructed using the mean hyperspectral signatures of the individual pathogenic strain/serovar from the reference library data.

A hierarchical clustering algorithm was also employed for classifying pathogenic strains/serovars at colony and cellular levels (Figure 7). At the colony level, the hierarchical clustering technique correctly grouped the similar strains/serovars of a given pathogen into clusters. At the colony level, six clusters were obtained when the dendrogram was cut at 0.44 (Figure 7a). The Big Six non‐O157 STEC serovars were grouped as one cluster and *E. coli* O157:H7 strains were grouped as a separate cluster (Figure 7a). Likewise, strains of *L. monocytogenes*, *S. aureus*, and *E. coli* O157:H7 were clustered into three different groups. The *Salmonella* serovars, Typhi, Infantis, and Senftenberg, were grouped together, whereas the remaining *Salmonella* serovars formed a different cluster (Figure 7a). On the other hand, the hierarchical clustering for the cellular‐level study had no definite pattern/trend and did not provide any meaningful grouping (Figure 7b).

## 4 DISCUSSION

The HSI technology has been studied to identify the colonies of Big Six non‐O157 STEC serovars (O26, O45, O103, O111, O121, and O145) on Rainbow agar plates using pure and mixed cultures (Yoon et al., 2013a, 2013b). Yoon et al. (2013a) utilized the HSI system for differentiating the colonies of non‐O157 STEC serovars using pure cultures. The hyperspectral data for colonies were acquired using a push‐broom line‐scan visible near‐infrared hyperspectral imager. The HSI system gathered the reflectance data for the wavelengths ranging from 368 to 1024 nm. The acquired data were analyzed using principal component analysis (PCA) in conjunction with Mahalanobis distance (MD) and *k*NN algorithms. The classification accuracies for serovars O111 and O121 were over 99%, whereas, for serovars O26, O45, O103, and O145, the classification accuracy varied between 84% and 100%. For the mixed‐culture study, using *k*NN, the classification accuracies for serovars O45, O11, and O121 were 100%. However, the classification accuracies for serovars O26, O103, and O145 were 95.80%, 88.54%, and 91.93%, respectively (Yoon et al., 2013b). The results from Yoon et al. (2013a) were comparable with our research, as the classification accuracies of non‐O157 STEC serovars O103, O111, and O45 in dairy products was 100%, and for serogroup O26, the classification accuracy was observed as 83.33%.

Windham et al. (2013) further expanded the spectral library of Big Six non‐O157 STEC serovars built by Yoon et al. (2013b). The hyperspectral images of the colonies on Rainbow agar plates were acquired in the wavelengths ranging from 400 to 1100 nm. The reference spectral library was constructed using pure cultures. After that, the ground beef was artificially inoculated with the respective serovars, and hyperspectral images of the colonies from the agar plates were acquired. The data were analyzed using a *k*NN classifier, where the classification accuracies for Big Six non‐O157 STEC serovars varied from 78.1% to 100%. The serovar O145 displayed the lowest classification accuracy of 78.1%, whereas, for serovars O45 and O111, the classification accuracy was 100% (Windham et al., 2013). Interestingly, in our research, the serovar O145 also exhibited the lowest classification accuracy of 66.67% in whole milk powder. Furthermore, Yoon et al. (2013a) observed that serovar O45 displayed the highest absorbance value in the visible spectrum (400–720 nm) compared to other serovars. The authors attributed this to the relatively dark appearance (low reflectance) of the O145 colony on the Rainbow agar. Likewise, in our research, the differences in the scattering intensities of *E. coli* O157:H7 strains and non‐O157 STEC serovars can be explained based on colony appearance. The *E. coli* O157:H7 colonies appeared cream, whereas non‐O157 STEC colonies were black. The hierarchical clustering also grouped O157:H7 strains and non‐O157 STEC serovars into two different clusters (Figure 7a). In addition, the *Salmonella* serovars Typhi, Infantis, and Senftenberg appeared white/cream compared to the black‐colored other *Salmonella* serovars. This fact was well supported by the hierarchical clustering algorithm, where the three *Salmonella* serovars mentioned above were grouped as a separate cluster compared to the cluster of the other seven *Salmonella* serovars. (Figure 7a).

Kammies et al. (2016) studied the use of near‐infrared HSI for differentiating colonies of Gram‐positive and Gram‐negative bacteria, pathogenic and non‐pathogenic bacteria, and similar species of the same genera. The bacteria colonies of *Bacillus cereus*, *E. coli*, *Salmonella* Enteritidis, *S. aureus*, and *S. epidermis* were isolated on Luria–Bertani agar plates. The hyperspectral images were acquired in wavelengths ranging from 920 to 2514 nm. After data collection, three different image mosaics or groups were created. Group 1 included *B. cereus*, *E. coli*, and *Salmonella* Enteritidis, as their colonies appeared white/cream. Group 2 had Gram‐positive bacteria (*B. cereus*, *S. aureus*, and *S. epidermis*), and group 3 encompassed *Staphylococcus* species. The data were analyzed using PCA, and partial least‐square discriminant analysis (PLD‐DA) models were employed to confirm the PCA data. For group 1, the classification accuracies for *B. cereus*, *E. coli*, and *S. epidermis* were 78.2%, 2.34%, and 21.3%, respectively. However, for groups 2 and 3, classification accuracy for individual bacteria varied from 82% to 99.96%. The authors attributed the differences in hyperspectral signatures to the variation in teichoic acid content, protein structures, and lipid content of bacteria. For example, a Gram‐positive bacterium has a thicker peptidoglycan layer embedded with proteins and teichoic acid (Schleifer & Kandler, 1972). On the contrary, the outer membrane of Gram‐negative bacteria is chiefly composed of lipopolysaccharides and proteins with an extremely thin peptidoglycan layer (Schleifer & Kandler, 1972). In our research, the differences in the hyperspectral signatures of Gram‐positive (*L. monocytogenes* and *S. aureus*) and Gram‐negative (*E. coli* and *Salmonella*) pathogens can also be attributed to the variation in the peptidoglycan layer and presence of proteins and teichoic acid.

The *Campylobacter* and non‐*Campylobacter* contaminant colonies on the same agar plate can appear visually similar and pose a significant challenge in identifying the desired pathogen. The HSI system at a macrolevel can also be used to differentiate the bacterial colonies. For example, Yoon et al. (2010) successfully differentiated *Campylobacter* colonies from non‐*Campylobacter* contaminants. Yoon et al. (2010) utilized *Campylobacter* species (*coli*, *lari*, and *jejuni*) and non‐*Campylobacter* contaminants (*Sphingomonas paucimobilis*, *Acinetobacter baumannii*, *Brevundimonas diminuta*, *Ochrobacterium* sp., and *Flavobacterium odoratum*) for building the standard reference library. The hyperspectral data for the bacterial colonies from blood agar or Campy‐Cefex agar plates were acquired in the wavelength range 400–900 nm. With the application of the band ratio algorithm, using two bands at 426 and 458 nm selected from continuum‐removed spectra of blood agar, the authors achieved a classification accuracy of 97%–99%.

Park et al. (2015) used an acousto‐optic tunable filter (AOTF)‐based hyperspectral microscopic imaging (HMI) system to classify *Salmonella* serovars (Enteritidis, Typhimurium, Heidelberg, Kentucky, and Infantis) and *Staphylococcus* species (*aureus*, *hemolyticus*, *hyicus*, *sciuri*, and *simulans*). The hyperspectral images were acquired at wavelengths from 450 to 800 nm with an exposure time of 250 ms and a gain value of 9. The authors used a dark‐field microscope with a metal halide as the illumination source and a spectral sweep mode for collecting contiguous spectral images. After image collection, the spectral image at 546 nm was used to select ROI from bacterial cells using ENVI software. The scattering intensity data for bacterial cells were collected and analyzed using R software. To identify the *Salmonella* serovars and *Staphylococcus* species, five different algorithms, including MD, *k*NN, linear discriminant analysis, quadratic discriminant analysis, and support vector machine, were used. The classification accuracy (using five different algorithms) varied from 99.55% to 100% for *Salmonella* serovars and 99.96%–100% for *Staphylococcus* species.

In another study, Park et al. (2012) utilized HMI to evaluate the spectral characteristics of the biofilms formed by *Salmonella* Enteritidis and STEC serotype O26 on food‐grade stainless steel coupons. The biofilms formed by *Salmonella* Enteritidis and STEC serotype O26 were immobilized on glass slides and analyzed under the HMI system. The hyperspectral images were acquired at 450 to 800 nm wavelengths at four different exposure times (50, 100, 250, and 500 ms) and three different gains (10%, 20%, and 40%). The authors discovered that the intensity for biofilm formed by *Salmonella* Enteritidis was distinct at 498, 522, 550, and 594 nm, whereas, for STEC serotype O26, the intensity at 546 nm was found to be distinct.

In our previous research, an affordable HSI system was assembled to identify single and mixed strains of *E. coli* O157:H7 and *L. monocytogenes* in dairy products (Unger et al., 2022). The HSI system was custom assembled by mounting a snapshot hyperspectral imager over a trinocular dark‐field microscope. Unger et al. (2022) used three strains of each pathogen, where Sorbitol MacConkey and PALCAM agars were utilized to isolate the colonies of *E. coli* O157:H7 and *L. monocytogenes*, respectively. The hyperspectral images at the cellular level were acquired at wavelengths ranging from 400 to 1100 nm, with 5 nm band intervals. Whole milk, cottage cheese, and cheddar cheese were used as the dairy products, inoculated individually with respective pathogens, and stored at 4°C for 24 h. Except for whole milk, the inoculated dairy products were individually diluted using 20 mL of 0.1% peptone water and stomached for 1 min. Afterward, the individual samples were streaked for isolation on the respective selective media, and the hyperspectral images at the cellular level were collected. The reference library was constructed using the images obtained from pure cultures, and pathogenic test cells from the inoculated dairy product samples were classified using *k*NN and cross‐validation techniques. Using this HSI system, *E. coli* O157:H7 and *L. monocytogenes* were classified with an accuracy level of 58.97% and 61.53% at the genus level, respectively. The results from Unger et al. (2022) are comparable to the results from this study for *E. coli* O157:H7, where an overall classification accuracy of 54.2% in dairy products was achieved.

The HSI system at the cellular level was employed by Michael et al. (2019) to classify four strains of *Cronobacter sakazakii*, five strains of *Salmonella* spp., eight strains of *E. coli*, and one strain of *L. monocytogenes* and *S. aureus*. The hyperspectral signatures of the bacterial cells were classified using PCA and the *k*NN classifier model, which were then validated using a cross‐validation technique. The classification accuracy of various strains within their respective genus (except for four strains) was 100%. When all strains of different genera were analyzed together, 100% classification accuracy was obtained for only five strains. Michael et al. (2019) also studied the effect of lauric arginate on the hyperspectral signatures of bacteria. They reported that lauric arginate‐treated cells of *C. sakazakii* BAA‐894, *E. coli* O157:H7, *Salmonella* Senftenberg, *L. monocytogenes*, and *S. aureus* had significantly different hyperspectral signatures than non‐treated healthy cells.

The light source employed affects the hyperspectral signatures of a given pathogen. Park et al. (2016) confirmed the possible variation in hyperspectral signatures when using different light sources. Park et al. (2016) used metal halide and quartz halogen light sources to obtain hyperspectral signatures of *Salmonella* serovars, Enteritidis, and Typhimurium. The wavelengths ranging from 450 to 800 nm at 4 nm band intervals, with an exposure time of 250 ms and a gain value of 9, were used to acquire the hyperspectral microscopic images. Brilliant Green Sulfa (BGS) and Xylose‐Lysine‐Tergitol4 (XLT4) agar plates were used as the selective media for isolating the *Salmonella* colonies. Thereafter, the bacterial cell suspension was prepared by mixing a few colonies from respective agar plates with filter‐sterile phosphate‐buffered saline (PBS). The bacterial slides were prepared using 10 μL of the bacterial suspension and immediately air‐dried in the biosafety cabinet for 15 min. The authors reported that spectral peaks for the serovars Enteritidis and Typhimurium were similar within the type of light source used. When metal halide was used as the light source, the spectral peaks for both serovars were observed at 458, 498, 546, 590, and 670 nm. On the other hand, the spectral peaks were obtained at 558, 646, 702, and 772 nm when halogen was used as the light source.

Therefore, a possible variation in hyperspectral signatures of bacteria across different research studies can be linked with the different light sources and hyperspectral imaging setups employed for acquiring the hyperspectral images. The selective media for isolating the bacterial colonies can also affect the hyperspectral signatures. The accuracy of various classification algorithms can be affected by the amount of reference or test data set. Therefore, a robust reference library should be constructed and updated regularly to achieve higher classification accuracies.

Although HSI is being studied for its application in rapid pathogen identification, the high initial cost (over $100,000) of pre‐assembled and pre‐programmed HSI systems has thwarted the full exploitation of this technology. The researchers can overcome the cost factor by custom assembling the HSI system using a hyperspectral camera that can meet their needs at a low cost. Our custom‐assembled HSI system is about five times cheaper than the commercially available pre‐programmed and pre‐assembled HSI setups (Unger et al., 2022). Additionally, using open‐source software to process and analyze the HSI data can help reduce the overall cost of the system.

In conclusion, the overall classification accuracy of 90.38% of the studied HSI system with the *k*NN classification model for various pathogens at the colony level supports the hypothesis that HSI can be used as a rapid pathogen identification tool in the food industry. To achieve the practical application of this HSI system, the reference library at the colony level should be made stronger by adding more hyperspectral data/replications to it. Moreover, this reference library can then be expanded to other foodborne pathogens as well as probiotic and spoilage bacteria. However, this HSI system was not effective in accurately identifying various pathogens at the cellular level. To improve the classification accuracy of this HSI system at the colony level, more research should be conducted to improve the magnification of bacterial cells under the microscope. To make this HSI system as a more rapid pathogen identification system, additional work will be done to shorten the enrichment and plating incubation times.

## AUTHOR CONTRIBUTIONS

**Amninder Singh Sekhon:** Data curation (lead); formal analysis (lead); methodology (equal); writing – original draft (lead). **Phoebe Unger:** Data curation (supporting); methodology (supporting); writing – review and editing (equal). **Sonali Sharma:** Data curation (supporting); methodology (supporting); writing – review and editing (equal). **Bhupinderjeet Singh:** Formal analysis (equal); writing – review and editing (supporting). **Xiongzhi Chen:** Formal analysis (equal); writing – review and editing (equal). **Girish M. Ganjyal:** Funding acquisition (supporting); project administration (supporting); writing – review and editing (equal). **Minto Michael:** Conceptualization (lead); data curation (equal); formal analysis (equal); funding acquisition (lead); investigation (lead); methodology (equal); project administration (lead); resources (lead); supervision (lead); writing – original draft (supporting); writing – review and editing (equal).

## CONFLICT OF INTEREST STATEMENT

The authors have no conflict of interest.

## Data Availability

Research data are not shared.
